# Contrast enhanced MRI and MR coronary angiography (MRCA) as one stop shop in patients with untreated myocardial infarction

**DOI:** 10.1186/1532-429X-11-S1-P280

**Published:** 2009-01-28

**Authors:** Carlo Liguori, Luigi Natale, Agostino Meduri, Antonio Bernardini, Riccardo Marano, Lorenzo Bonomo

**Affiliations:** 1 Università Cattolica del Sacro Cuore – Policlinico A. Gemelli, Rome, Italy; 2 "G. Mazzini" Hospital – ASL Teramo, Teramo, Italy

**Keywords:** Coronary Angiography, Short Axis, Conventional Coronary Angiography, Signa Excite, Blinded Reader

## Purpose

To define MRI role in patients with myocardial infarction not treated by PCI.

## Methods and materials

24 pts (17 males, 7 females) with myocardial infarction (7 anterior, 9 lateral, 8 inferior), untreated by PCI, underwent MRI within three weeks after infarction. MRI was performed on a 1.5 T scanner (GE Signa Excite) with this protocol:

1) Triple-IR FSE sequence short axis for edema;

2) Fiesta sequences on short, horizontal and vertical long axes, for regional and global systolic function;

3) FGR-ET short axis sequence for first pass;

4) IR-FGRE sequence on short, horizontal and vertical long axes, for infarct size.

5) Before delayed imaging, MRCA with breath-hold 3D Fiesta VCATS technique was obtained. MRCA was compared with the gold standard conventional coronary angiography (CA), performed within 24–48 h.

## Results

MRCA was scored with a 3 points scale (0 = good, 1 = sufficient, 2 = poor) by 2 blinded readers; 3 exams were excluded as pts could not hold their breath. MRCA showed 11 severe stenoses (>50%) and 9 occlusions, with 1 exam not evaluable for poor quality; CA showed severe stenoses in 14 and 7 occlusions. Collaterals and retrograde filling were present in 2 occlusions at CA; in these 2 cases DE at MRI was ≤50%. DE and FP defects distribution always showed correlation with the diseased vessel. MRCA showed 100% sensitivity, 78.6% specificity, 70% PPV, 100% NPV.

## Conclusion

In pts not treated by primary or rescue PCI, MRCA can rule out occlusion and/or significant stenosis; the correlation with segmental distribution of DE is excellent.

